# Wearable Multispectral Sensor for Newborn Jaundice Monitoring

**DOI:** 10.3390/s25237293

**Published:** 2025-11-30

**Authors:** Fernando Crivellaro, Ana Isabel Sousa Pedroso, Anselmo Costa, Pedro Vieira

**Affiliations:** 1 Department of Physics, Faculty of Science and Technology, NOVA University of Lisbon, Caparica Campus, 2829-516 Caparica, Portugal; f.crive@gmail.com (F.C.); anaisabelsousa.92@gmail.com (A.I.S.P.); 2 Department of Pediatrics, Hospital Garcia de Orta, EPE, 2805-267 Almada, Portugal; acosta@hgo.min-saude.pt

**Keywords:** jaundice, bilirubin, newborns, non-invasive, wearable sensors, multispectral sensor, optical sensor

## Abstract

Newborn immaturity transcends their bodies, immune systems, and communication and perception capabilities, making them vulnerable to the environment. Neonatal jaundice is a common condition, with higher levels of unconjugated bilirubin concentration having neurotoxic effects. Newborns are routinely monitored visually or non-invasively with transcutaneous bilirubinometry (TcB) due to their biological immaturity to conjugate bilirubin. Higher levels of bilirubin are a sign that there is either an unusual rate of red blood cells breaking down or that the liver is not able to eliminate bilirubin through bile into the gastrointestinal tract. Actual devices used in bilirubin screening are hand-held and do not allow operation outside the hospital. Based on these factors, a continuous bilirubin monitoring device for newborns was developed, which enables the evaluation of neonatal jaundice inside or outside the hospital. This non-invasive device operates through a mini-spectrometer in the visible range. It was calibrated with phantoms, and its operation was compared with a gold-standard bilirubinometer through in vitro experiments, exploring the practical range of bilirubin variation in newborns and presenting a clinically acceptable deviation of 1 mg/dL. These experiments showed that the continuous bilirubin monitoring device developed has the potential to be used for remote monitoring of jaundice in newborns.

## 1. Introduction

**Figure 1:**
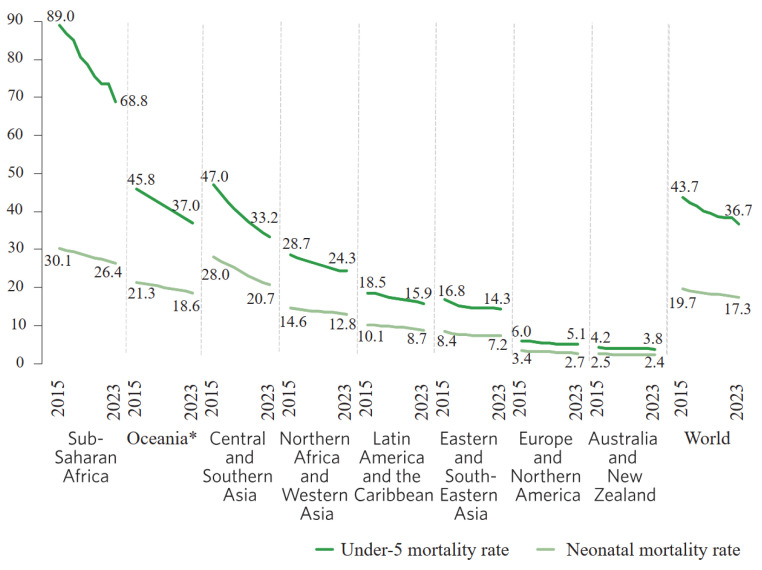
Neonatal mortality rate for children under 5 for 2015–2023 (deaths per 1000 live births). * Excluding Australia and New Zealand [1].

A global partnership made by United Nations country members established 17 Sustainable Development Goals (SDGs) for the 2030 Agenda, and Sustainable Development Goal 3 is an urgent call for action to “ensure healthy lives and promote well-being for all ages”. This goal was broken down into nine associated targets, one of which is to end all preventable deaths in children under 5 years of age, with all countries aiming to reduce neonatal mortality to at least as low as 12 per 1000 live births. However, despite the neonatal mortality rate dropping to 17 deaths per 1000 live births in 2023, a 44% reduction from the 31 deaths recorded in 2000, this indicator has presented slower progress since 2015. As presented in Figure 1, a reduction of only 16% was recorded between 2015 and 2023. Consequently, 65 countries need faster reduction rates in order to achieve the SDG target and, therefore, to prevent around 8 million neonatal deaths [1].

Exploring the above-mentioned data in more depth, the Global Burden of Disease study by the Institute for Health Metrics and Evaluation (IHME) conducted the largest and most comprehensive effort to quantify health loss across places and over time, so that health systems can be improved and disparities eliminated. The study ranked “neonatal hemolytic” among the top 10 causes of neonatal deaths in the world in 2021. The term “neonatal hemolytic” refers to elevated Total Serum Bilirubin (TSB), as manifested by extreme hyperbilirubinemia (TSB > 25 mg/dL) and kernicterus (bilirubin-induced brain injury) [2].

**Figure 2:**
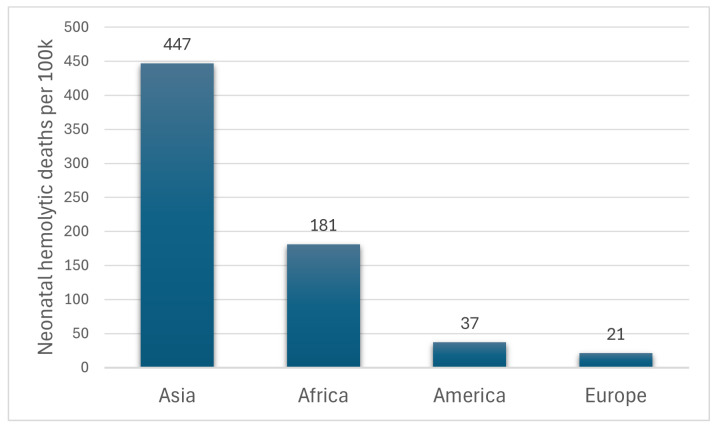
Deaths by neonatal hemolytic per 100,000 live births for 2021 [2].

The data from the Global Burden of Disease study in 2021 relates to hospital-based information only; that is, it may be an underestimation considering the high rates of out-of-hospital births in low-resource countries. Even so, this ranking highlights Africa and especially Asia as having the highest neonatal hemolytic death burdens, which were also reported by other studies [3], as presented with statistics in Figure 2. The underlying causes include the high prevalence of Glucose-6-Phosphate Dehydrogenase (G6PD) deficiency, an inability of caregivers to promptly identify jaundice, a lack of or ineffective phototherapy, and unreliable access to bilirubin estimation [4].

Based on the above-mentioned scenario, neonatal hemolytic-related mortality rates still negatively impact society. In addition, high bilirubin levels in newborns are a critical and real issue. Therefore, it is clear that improvements in jaundice management, which is the main objective of this work, can directly impact the estimate of deaths for the 2030 SDGs, as will be discussed in the next sections.

### Jaundice Management

Jaundice is an abnormal yellowing of the skin, sclerae, or mucous membranes due to the accumulation of bilirubin in these tissues [5]. According to epidemiology data, jaundice incidence is related to different causes, being more frequent in certain age groups. Neonatal jaundice, more specifically, occurs at rates of 50% for term newborns and 80% for preterm newborns. These significant rates, and the possibility of evolution to encephalopathy or death, lead to a strong recommendation to routinely monitor newborns for the development of jaundice [6].

The first and obvious technique for jaundice detection is a visual inspection of the newborn. Although visual inspections are recommended as the first patient approach, they are classified as not reliable for estimating bilirubin levels in newborns. Thus, bilirubin should be measured non-invasively by transcutaneous bilirubinometers or invasively with serum bilirubin analyses obtained from blood samples, which is a time-consuming and stressful procedure for newborns [7,8]. Transcutaneous bilirubinometry (TcB) is the focus of this work, as it is a reliable and non-invasive method that can reduce the number of blood samples required for jaundice evaluation, or even eliminate the need to collect them. However, it can still be improved in terms of device operation and measurement periodicity, as well as in better correlating serum and transcutaneous bilirubin [9,10,11,12].

Essentially, TcB measurements involve analyses of the skin’s diffuse reflectance when it is exposed to a light source with different wavelengths. The spectral content of measured light depends on the concentration of the different chromophores (e.g., melanin, hemoglobin, and bilirubin) in the skin and subcutaneous tissues. Therefore, through absorption spectral differences, the TcB level can be calculated [13,14]. However, even considering TcB as a practical approach for jaundice management, it requires a trained technician to operate the bilirubinometer and, consequently, measurements are performed within hospital facilities. This constraint can be considered a burden for the hospital in patient management, as there are cases in which the baby and mother need to be present only for bilirubin monitoring. This is one of the possible improvements and also one of the motivations of this work. A remote bilirubin monitoring device could improve the family’s quality of life at this critical moment and optimize hospital allocation, particularly when bilirubin levels return to normal without medical intervention or newborn injury.

Also, as jaundice management is carried out over time, studies reinforce the analysis of the rate of rise in bilirubin as a predictor of risk designation, an indicator of phototherapy timing and duration, and even as support for early-discharge policy in term and late-preterm neonates [11,15,16,17]. Increased bilirubin production coupled with co-inheritance of G6PD can result in an increase in bilirubin rate exceeding 0.2 mg/dL per hour. Delaying the recognition of this clinical scenario due to the inability to adequately monitor this rapid progression, together with limited therapeutic options to reverse it, can lead to adverse lifelong consequences in some infants or even kernicteric mortality [18,19]. Still considering the bilirubin rate of rise as a relevant clinical feature, the study by Joseph Chou [20] developed ML models to predict subsequent bilirubin measurements and provide advanced clinical decision support. Hence, a continuous bilirubin monitoring device can enhance the assumptions of these studies and probably provide new insights into newborn jaundice management, since it could be correlated with other vital signs, such as heart rate, respiratory rate, and temperature. For example, there are cases where hypothermia presents a significant relationship with respiratory distress, as well as with jaundice [21,22,23].

Another important consideration is the elevated prevalence of neonatal hyperbilirubinemia in Low- and Middle-Income Countries (LMICs), which is primarily attributable to the relatively low rate of hospital-based childbirths within these regions. A significant portion of deliveries takes place outside healthcare facilities, leading to the imperative for mothers and families to assume responsibility for recognizing jaundice in neonates through visual assessment, which has proven to be inaccurate [24,25]. In this case, it is clear that the Internet of Things (IoT) and sensing technologies could extend remote bilirubin monitoring to these newborns by detecting and addressing critical cases of hyperbilirubinemia before any adverse consequences for the newborns [26]. This work aligns with that idea, as it aims to develop a wearable device for continuous bilirubin monitoring, which can be used inside or outside the hospital.

## 2. Materials and Methods

In this section, the developed solution is described, along with the algorithm used to estimate bilirubin concentration from optical measurements, as well as the calibration procedure and the methodology for comparison with a commercial bilirubinometer.

### 2.1. The Integrated Solution

Although bilirubinometers are hand-held and perform analysis considering multiple wavelengths in the visible spectrum, wearables are portable and generally analyze one or two wavelengths [27,28]. So, both device types were merged into one solution, enabling this work’s approach of continuous bilirubin monitoring and opening the possibility for future integration with other sensors like photoplethysmography (PPG), respiratory rate, and temperature sensors through wireless communication in the vicinity of the human body, called a Wireless Body Area Network (WBAN), which was investigated in a previous work [29]. WBAN networks operating in this short-range area enable connectivity between this and other wearable devices without posing any hazard to the newborn’s health.

**Figure 3:**
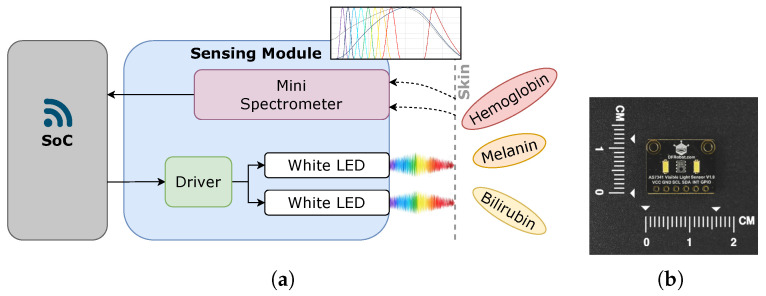
(**a**) Prototype diagram. (**b**) AS7341 sensing module.

The developed prototype, called *ProtoBili*, is shown in Figure 3a, and its characterization was reported in a previous study [30], which also details the prediction of the device’s placement status as a signal-quality indicator. Its sensing module is composed of the AS7341 mini-spectrometer from ams OSRAM with 9 channels, which is a low-power, high-precision, multi-channel light sensor. With an operating range from 415 to 680 nm, plus one extra channel at 910 nm, it performs acquisitions with a resolution of 16 bits and has a half cone angle of 40 degrees, as shown in Figure 3b.

On each side of the sensor, two cool white LEDs with a 120-degree viewing angle serve as light sources, with a peak intensity at 440 nm and an extended spectrum from 540 nm to 680 nm. LED driving is also handled by the AS7341, which has one pin for light-source control. The wireless device connectivity is handled by the System-on-Chip (SoC) ESP32, which interfaces with the AS7341 sensor board through a two-wire serial communication protocol, $I^{2}C$.

**Figure 4:**
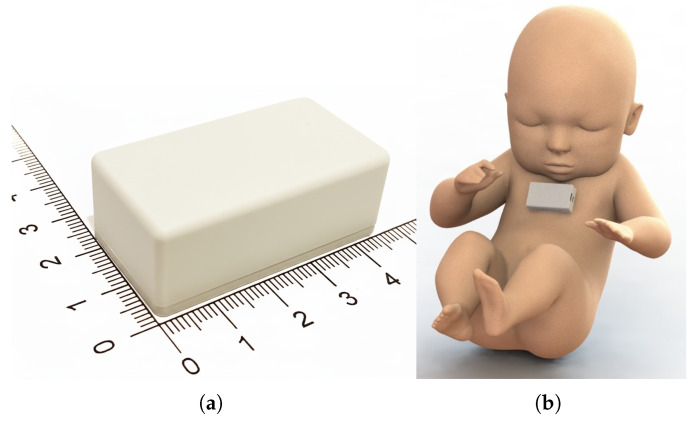
Bilirubinometer prototype, called ProtoBili: (**a**) Prototype size (cm). (**b**) Device positioned on a newborn’s sternum.

To address future deployments in newborns, the device was designed with a small form factor. Figure 4a shows the size of the ProtoBili, which was designed to be placed on the newborn’s sternum or forehead and perform non-invasive TcB measurements, as shown in Figure 4b. The device communicates with a gateway via a BLE network, as it was found to be the most suitable short-range wireless technology for this application when safety, reliability, and performance are considered. BLE technology supports multiple topologies, including star, point-to-point, and mesh. In addition, it can achieve data rates up to 2 Mbps and transmit at a power as low as 10 μW [29].

### 2.2. Light and Skin Relationship

The interaction of light with biological tissues is a complex process involving multiple optical effects, such as reflection, refraction, absorption, and scattering. Biological tissues are optically inhomogeneous and absorbent media whose average refractive index is higher than that of air. As Snell’s law states, when light encounters a boundary between two media, a partial reflection occurs at the tissue–air interface, while the remaining light penetrates the tissue. Together, absorption and multiple scattering cause light beams to broaden and decay as the photons travel through tissue [31,32].

The light absorption of tissue depends on its molecular composition. The absorption coefficients of different tissue components influence light penetration at specific wavelengths. This behavior is partially described by the *Beer–Lambert Law* [33]:

(1) $$I\left(x\right)=I_{0}e^{-\mu_{a}x}$$

where I(x) is the intensity at distance *x* into the tissue, $I_{0}$ is the intensity of the incident light, and $\mu_{a}$ is the absorption coefficient of the material.

Suppose there are N different types of absorbing molecules in a tissue, and let the concentration of the nth molecule type be $c_{n}$ (M/L). Let its molar absorption coefficient (or molar extinction coefficient) be $\varepsilon_{n}\left(\lambda \right)$ ($M^{-1}{\mathrm{cm}}^{-1}$). The absorption coefficient is given by [34]

(2) $$\mu_{a}=\sum_{n=1}^{N}c_{n}\varepsilon_{n}\left(\lambda \right)$$

In addition to absorption, other important light–tissue interactions occur in the skin, which affect the optical path of the incident light. Both forward scattering and backscattering of incident light within tissue are used in a variety of biophotonics applications, and the degree of forward versus backward scattering is related to the anisotropy factor in the reduced scattering coefficient. The reduced scattering coefficient, $\mu_{s}^{\prime }$, is defined as [34]

(3) $$\mu_{s}^{\prime }=\mu_{s}\left(1-g\right)$$

where $\mu_{s}$ is the scattering coefficient and *g* is the anisotropy factor, which is the average cosine of the scattering angle. The reduced scattering coefficient is a measure of the scattering of light in a medium, taking into account the anisotropy of the scattering process.

Histological samples from neonates were evaluated using a videometric image analysis system [35]. The scattering measurements were related to a combination of Mie scattering by the collagen fiber bundles in the dermis and Rayleigh scattering by smaller particles. It was concluded that scattering increases with age, apparently due to the increase in size and concentration of collagen fiber bundles. Therefore, a relationship was established between scattering, gestational maturity, and wavelength, as presented in Equation (4):

(4) $$\mu_{s}\left(1-g\right)\left(\lambda \right)=y_{int}\left(\lambda \right)+m\left(\lambda \right)\cdot maturity$$

where $\mu_{s}\left(1-g\right)\left(\lambda \right)$ is expressed in cm^−1^ and maturity is expressed in weeks. The empirical fit of the y-intercept as a function of the wavelength is detailed in Equation (5):

(5) $$y_{int}\left(\lambda \right)=22.5-0.14267928\cdot \lambda +0.000129357\cdot \lambda^{2}$$

while the slope, *m*, is given by Equation (6):

(6) m(λ)=2.978−0.0029985·λ

In 1962, Twersky introduced the idea of supplementing the *Beer–Lambert Law* with light-intensity losses due to scattering [36]. This approach, reinforced by Anderson et al. [37] and improved by Delpy et al. [38], introduces the concept of optical density (OD), which, in comparison to absorbance, considers both absorption and scattering [34,39]. The study by Steven Jacques [40] confirmed, from Monte Carlo simulations and biopsies of newborns’ skin, that direct analysis of reflectance cannot be applied to accurately determine absorption in tissue. The suggestion is that the relationship between optical density and absorption in a tissue is not linear. Therefore, the use of linear regression analysis to correlate bilirubin concentration with changes in skin optical density will not perform well in populations where skin scattering properties and blood concentration vary.

Based on the approach adopted in the above-mentioned studies, the distance *x* from Equation (1) becomes the effective optical path length, $L_{eff}$, which accounts for tissue scattering. Another factor, *G*, represents the influence of the optical collection efficiency, since the receptor’s reflectance measurement cannot collect all the light reflected from the skin. These assumptions enable accurate absorption determination for jaundice evaluation in newborns [38,40]. By encompassing the above-mentioned aspects, the OD can be expressed as

(7) $$OD\left(\lambda \right)=-ln\left(\frac{I}{I_{0}}\right)=L_{eff}\cdot \mu_{a}+G$$

$L_{eff}$ was found to be directly dependent on the penetration depth of light in the tissue (δ) and, therefore, inversely dependent on both $\mu_{a}$ and $\mu_{s}^{\prime }$ [33,35]:

(8) $$L_{eff}\propto \delta \propto 1/\sqrt{\left(\mu_{a}+\mu_{s}^{\prime }\right)}$$

However, while Equation (7) considers infinite-thickness skin, the real reflectance measurement is taken over finite-thickness skin. This implies the addition of an extra correction factor *c* in the analysis:

(9) $$OD\left(\lambda \right)=L_{eff}\cdot \mu_{a}+G+c$$

The effect of the skin-correction factor *c* was detailed in research by Steven Jacques on newborn skin [40], and it is related to the newborn’s maturity. Two equations were established for *c*: one for the green region, Equation (10), where there is no influence from the bilirubin chromophore:

(10) $$c_{g}=1-0.27\cdot e^{-0.057\cdot maturity}$$

and another for the blue region, Equation (11), where skin, blood, and bilirubin are the main actuating chromophores:

(11) $$c_{b}=1-0.26\cdot e^{-0.088\cdot maturity}$$

Based on the theoretical framework presented above, the specific aspects of the algorithm for bilirubin characterization from skin spectra are presented in the next section and are also applicable to bilirubin characterization from skin-related phantom spectra measurements.

### 2.3. Bilirubin Calculation Algorithm

To accommodate skin variations in the diverse newborn population, it is imperative that the scattering properties of a neonate’s skin be determined regardless of melanin pigmentation. As shown in Equation (4), the scattering increases with age (maturity) as collagen fiber bundles increase in size and concentration. On the other hand, there is no obvious dependence of $\mu_{a}$ on age.

**Figure 5:**
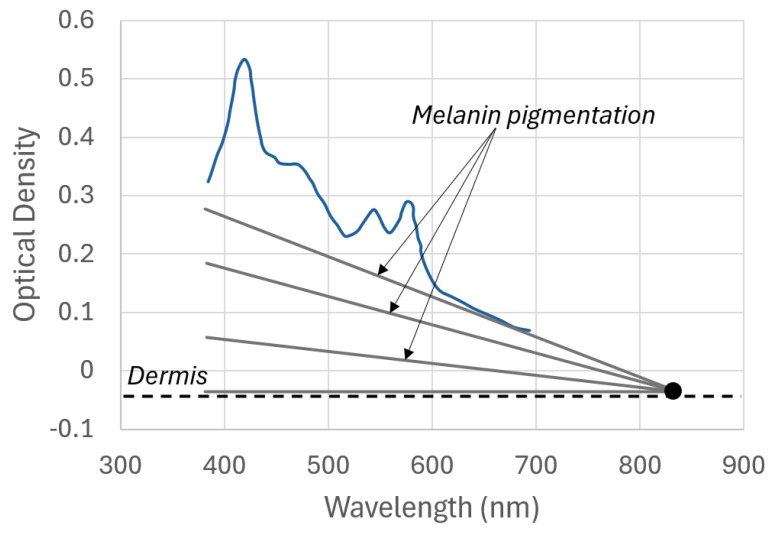
Influence of multiple melanin pigmentation levels on the skin spectra.

Therefore, maturity identification from skin is performed by linearly extrapolating the reflectance measurements from the red region (specifically, 630 nm and 680 nm) up to 837 nm ($M_{837}$). The reflectance at this point (see Figure 5) is invariant with melanin pigmentation and depends only on the scattering properties of the skin [35]. The equivalent measurement at 837 nm ($M_{837}$) is empirically related to maturity (in weeks) according to Equation (12) [40]:

(12) $$M_{837}=2.43\cdot log\left(maturity\right)-2.72$$

where $M_{837}$ is the extrapolated reflectance measurement at 837 nm.

By knowing the maturity of the newborn, the reduced scattering coefficient is determined for each wavelength using Equation (4), which is then used for the calculation of the effective optical path length ($L_{eff}$) in Equation (8). The maturity also enables the calculation of the skin-correction factors (*c*) using Equations (10) and (11).

Besides the above relationship with maturity, reflectance at 837 nm also relates to the melanin content in the skin [41]. The epidermal melanin acts as an attenuation filter for light entering and exiting the skin, but it has little effect on the reflectance and spreading of light in the dermis [32]. The melanin absorption spectrum decreases linearly with wavelength in the visible region, and its optical density measurement has a pivot point at around 837 nm [35]. Therefore, because the skin spectrum above 600 nm is less influenced by blood and bilirubin, a straight line can be fit to two points above 600 nm and considered representative of the melanin effect in the OD. This melanin effect is then removed from the OD spectrum, allowing analysis of the remaining chromophores.

For identifying and quantifying the skin chromophores, after subtracting the melanin effect, the next step is to calculate the absorption and scattering within the tissue, which can be performed iteratively. Assuming a predetermined scattering, the absorption is adjusted until a predicted measurement reflectance matches the real measured reflectance [40].

Assuming an optical density spectrum without the melanin effect, the next step is to identify the influence of the remaining absorbers. At this point, two chromophores, in addition to melanin, still affect the spectrum in the green region: blood and skin. In the blue region, the spectrum is influenced by blood, skin, and bilirubin. The skin absorption coefficients for newborns, determined by [40], for the green and blue spectral regions are, respectively,

(13) $$\mu_{aSkin}^{g}=0.81\;{\mathrm{cm}}^{-1}$$

(14) $$\mu_{aSkin}^{b}=1.71\;{\mathrm{cm}}^{-1}$$

**Figure 6:**
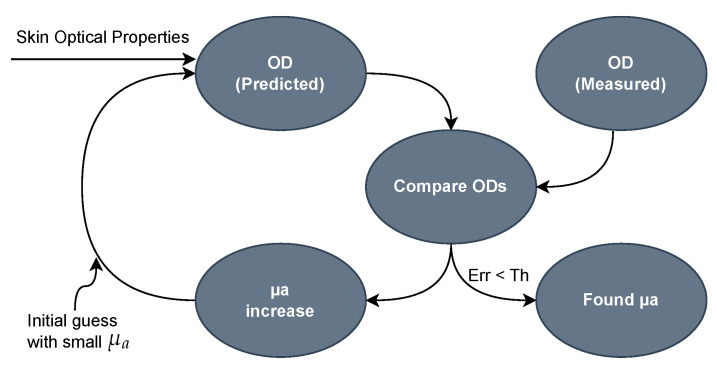
Iterative evaluation of the absorption coefficients after removing the melanin effect.

The resultant absorption coefficients for the green, $\mu_{a}\left(\lambda_{g}\right)$, and blue, $\mu_{a}\left(\lambda_{b}\right)$, spectral regions are calculated according to the iterative process presented in Figure 6, which was adapted from [40]. This iterative process starts with a small initial guess for $\mu_{a}$, which is then aggregated to the skin optical properties (maturity, skin-thickness correction) in Equation (9) to calculate the predicted optical density ($OD_{PREDICTED}$). The $OD_{PREDICTED}$ is compared with the measured optical density ($OD_{MEASURED}$) and, if the difference (Err) is lower than a specified threshold (Th), the iteration process stops, and the determined value of $\mu_{a}$ is used for subsequent phases. Otherwise, $\mu_{a}$ is increased by a maximum step of 1%, and another iteration is initiated. The maximum number of iteration cycles is preset to avoid infinite loops if convergence is not achieved.

After the iteration for the green region and determination of its absorption coefficient, considering the skin absorption coefficient from Equation (13), the blood absorption coefficient is determined using Equation (15):

(15) $$\mu_{aBlood}\left(\lambda_{g}\right)=\mu_{a}\left(\lambda_{g}\right)-\mu_{aSkin}^{g}$$

Then, assuming a successful iterative process for the blue region, and considering the skin absorption coefficient from Equation (14) and the blood absorption coefficient from Equation (15), the bilirubin absorption coefficient is determined using Equation (16):

(16) $$\mu_{aBilirubin}\left(\lambda_{b}\right)=\mu_{a}\left(\lambda_{b}\right)-\mu_{aBlood}\left(\lambda_{b}\right)-\mu_{aSkin}^{b}$$

where $\mu_{aBlood}\left(\lambda_{b}\right)=k\cdot \mu_{aBlood}\left(\lambda_{g}\right)$ and *k* was predetermined by [40] as 1.4.

Finally, the bilirubin molar concentration is calculated using Equation (17):

(17) $$Cm_{Bilirubin}=\mu_{aBilirubin}\left(\lambda_{b}\right)/\varepsilon_{Bilirubin}\left(\lambda_{b}\right)$$

To convert to the usual mg/dL format, Equation (18) is used:

(18) $$C_{Bilirubin}=Cm_{Bilirubin}\cdot Mm_{Bilirubin}\cdot 100$$

where $Mm_{Bilirubin}$ = 584.66 g/mol is the approximate molar mass of bilirubin.

### 2.4. Calibration Procedure

As shown in the previous section, the jaundice evaluation developed in this work is based on bilirubin concentration analysis. The bilirubin concentration impacts the absorption coefficient presented in Equation (2). The absorption coefficient is related to the prototype measurements according to Equation (9), which is an adaptation of Equation (7).

The missing parameters from Equation (9), $L_{eff}$ and *G*, were determined through measurements performed on phantoms. By controlling the phantom composition, the optical properties at specific desired wavelengths were fine-tuned to levels similar to those of the simulated tissue [42,43]. The phantoms were developed based on previous expertise and on the literature, which describes the main chromophore variations and their influence on the skin’s optical response [14,44,45]. The phantom’s base is agarose, and the scattering element is Intralipid (SMOFlipid ^®^, Fresenius Kabi, Portugal, 200 mg/mL) [42]. All phantoms were prepared considering a final volume of 100 mL, since they were deposited in specific containers developed for this purpose. These containers were built by additive manufacturing from black Polylactic Acid (PLA) to minimize their influence on the reflectance measurements.

Considering a skin-correction factor, along with discussions in the literature about the effect of skin tone on TcB measurements [32,45], multiple phantoms were constructed, simulating similar blood contents across three skin tones and four categories in which the bilirubin concentrations were varied. The spectral absorption in the blue region (e.g., 460 nm) was kept as similar as possible across the phantoms to mimic a stable cutaneous hemoglobin concentration. Phantom groups with similar synthetic blood concentrations but three different melanin pigmentation levels (brown, medium, and fair-skin equivalent) were created. The bilirubin concentration was varied equally in each skin tone group (0.5 mg/dL, 1.5 mg/dL, 2.5 mg/dL, and 5.0 mg/dL), keeping simulated melanin concentrations similar within each group and simulated blood concentrations similar among all phantoms. The synthetic blood absorption coefficient was based on a normal human hemoglobin concentration of 150 g/L [46].

Then, a calibration procedure was established to determine $L_{eff}$ and *G*. This procedure was based on the theory presented in Section 2.2 and Section 2.3 and assumed that the developed phantoms provided a sufficient approximation of the main skin effects detailed above. The calibration steps were as follows:

- The spectra were extracted for each phantom and evaluated with respect to their melanin content;
- The melanin effect was removed from the spectra;
- What remained was the influence of blood, bilirubin, and skin in the blue range (460 nm), as presented in Equation (19);
- The effective optical path length $L_{eff}$ and the collection efficiency factor *G* were then determined pairwise by solving Equations (20) and (21) among all the phantoms, with the average value then taken as the initial guesses for *G* and $L_{eff}$;
- Finally, the values of $L_{eff}$ and *G* were fine-tuned to achieve the best match for the predetermined bilirubin concentration levels of the phantoms.

(19) $$\mu_{a}{\left(460\right)}_{Cx}=\mu_{aBilirubin}{\left(460\right)}_{Cx}+\mu_{aBlood}\left(460\right)+\mu_{aSkin}\left(460\right)$$

(20) $$OD{\left(460\right)}_{C1}=L_{eff}\left(460\right)\cdot \mu_{a}{\left(460\right)}_{C1}+G+c_{460}$$

(21) $$OD{\left(460\right)}_{C2}=L_{eff}\left(460\right)\cdot \mu_{a}{\left(460\right)}_{C2}+G+c_{460}$$

The effective optical path length obtained from the calibration was $L_{eff}=0.22/\sqrt{\left(\mu_{a}+\mu_{s}^{\prime }\right)}$, and G=−2.195. These values were integrated into the bilirubin calculation algorithm.

### 2.5. Comparison with Commercial Bilirubinometer

**Figure 7:**
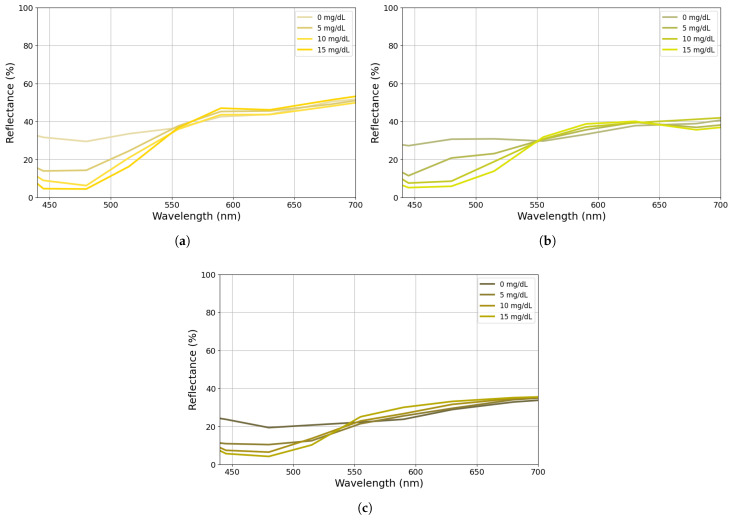
Phantom spectra for different skin-tone simulations: (**a**) Fair skin tone. (**b**) Moderate skin tone. (**c**) Brown skin tone.

To demonstrate the concept of using the AS7341 mini-spectrometer for jaundice monitoring, three groups with different skin-tonality phantoms were constructed for an in vitro experiment. Their construction followed the same approach as the phantoms built for the calibration procedure but with different bilirubin concentrations across groups. For this experiment, the selected bilirubin concentrations were 0 mg/dL, 5 mg/dL, 10 mg/dL, and 15 mg/dL, which correspond to the most common levels for newborns in the first days of life [47]. The idea of this phantom set was to evaluate the capacity of the developed prototype to identify different bilirubin levels in different skin tones, since this is one of the main problems with current commercial bilirubinometers [45]. Figure 7 exemplifies the reflectance spectra of the three phantom groups with different skin tones and bilirubin concentrations acquired by the AS7341 mini-spectrometer in the ProtoBili. The differences between the samples within each skin tone group and bilirubin level were below 8.5%, with an average variation of 3.5%.

The analysis of the bilirubin concentration was performed using the ProtoBili measurements over these phantoms with the algorithm detailed in Section 2.3 and Section 2.4. The results were compared with the measurements obtained with a commercial bilirubinometer, the JM-105 from Dräger, which is one of the most widely used devices in hospitals [48]. The devices were statistically compared through Bland–Altman analysis and Lin’s concordance correlation coefficient (CCC).

**Figure 8:**
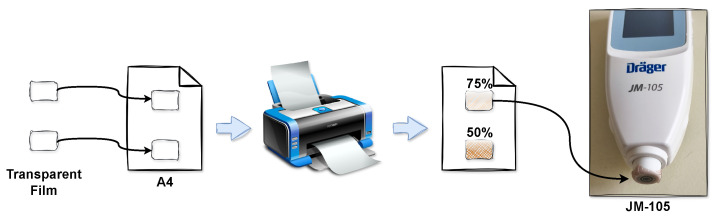
Commercial bilirubinometer setup.

The JM-105 operates using an optical differential path technique. This approach compares a short optical path, which captures reflected/scattered light from the shallow subcutaneous tissue, with a long optical path, which captures light from the deep subcutaneous tissue. Therefore, the JM-105 returns a zero measurement for uniform phantoms, such as those developed in this work. To overcome this limitation, a translucent film with a similar thickness to a newborn epidermis was placed over the bilirubinometer tip, enabling the JM-105 measurements. By using the same approach as [45], two transparent films (Hartmann-Hydrofilm, reference 970 004) were fixed in a sheet of paper, and then the desired films were printed on them. As shown in Figure 8, two films were tested, with 75% and 50% translucency, which were applied to the bilirubinometer tip before measuring the phantoms [45,49].

## 3. Results and Discussion

Measurements of the phantoms with the commercial bilirubinometer, as well as with the ProtoBili prototype, were acquired for each phantom group. The results are presented as box plots, where the box shows the data quartiles, and the whiskers extend to the rest of the distribution. Then, a Bland–Altman analysis was used to statistically compare the devices’ readings with predefined phantom levels (0 mg/dL, 5 mg/dL, 10 mg/dL, and 15 mg/dL).

### 3.1. Bilirubinometer: JM-105

**Figure 9:**
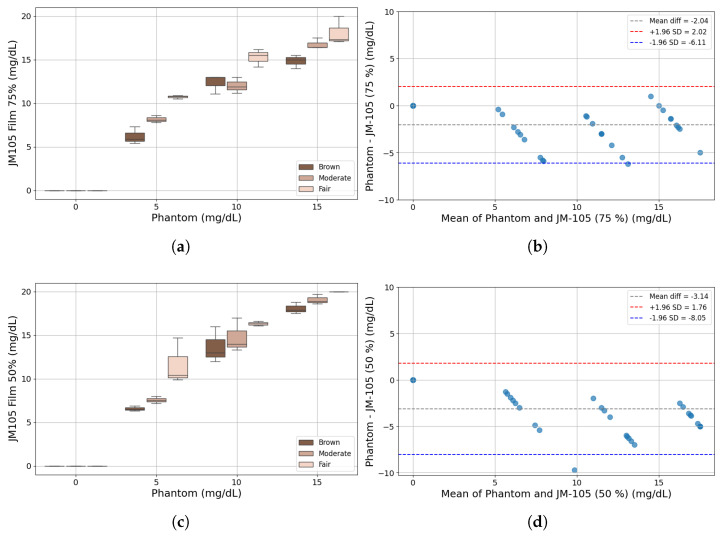
Prototype measurements of phantoms: (**a**) Bilirubin concentration per phantom with 75% translucent film. (**b**) Bland–Altman analysis against the respective phantom concentration level (75% translucent film). (**c**) Bilirubin concentration per phantom with 50% translucent film. (**d**) Bland–Altman analysis against the respective phantom concentration level (50% translucent film).

Figure 9a shows the results for the JM-105 measurements with 75% film translucency for phantoms with different skin tones and bilirubin levels. The brown-tone phantoms exhibit measurements with a mean value lower than those of the fair-tone phantoms, while most measurements, regardless of tone, show higher concentrations than the predefined phantom levels. This is clearly shown in Figure 9b, where the Bland–Altman analysis shows a mean difference of −2.04 mg/dL, with a variation of ±4.07 mg/dL, considering a confidence interval of 95%.

However, with a less translucent film (50%), the JM-105 increased the difference between its measurements and the predefined phantom levels, as illustrated in Figure 9c and statistically depicted in Figure 9d. In this case, the mean difference between them was −3.14 mg/dL, with a 95% confidence interval of ±4.91 mg/dL. These values are higher than the accuracy of ±2.3 mg/dL specified in the manufacturer’s datasheet [48].

### 3.2. Bilirubinometer: ProtoBili

**Figure 10:**
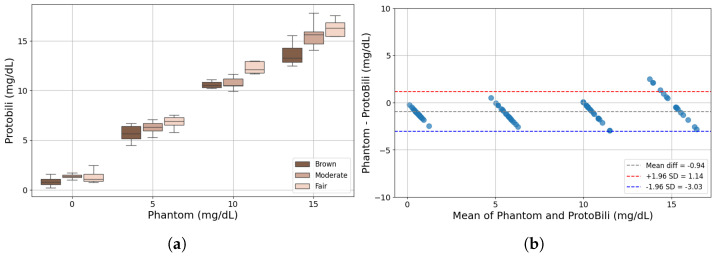
Prototype measurements of phantoms: (**a**) Bilirubin concentration. (**b**) Bland–Altman analysis against the respective phantom concentration level.

The measurements of the ProtoBili are presented in the same way as the JM-105 bilirubinometer. Figure 10a illustrates the device performance for the phantoms with different tones, and, similar to the JM-105 measurements, the brown-tone phantoms exhibit lower mean bilirubin concentration values in comparison to the fair-tone phantoms. Also, in general, the ProtoBili measurements are higher than the predefined phantom levels.

Figure 10b presents a statistical visualization of the Bland–Altman analysis. The mean difference between the predefined phantom levels and the ProtoBili was −0.94 mg/dL, with a variation of ±2.09 mg/dL, considering a confidence interval of 95%.

The ProtoBili mean difference was lower than what was seen in the JM-105 measurements with both the 75% and 50% translucent films. Also, comparing the measurement variations between the devices, the ProtoBili presented better precision. The first can be explained by the fact that these devices use different measurement techniques, and the JM-105’s use of the films could be affecting the results. However, regarding variations, the differences may be due to measurement procedures, which can negatively affect signal quality [50,51,52].

As the JM-105 is held in one hand and requires pressure against the phantom to trigger the measurement, it leaves more room for different angles and phantom deterioration after each sampling. Also, the translucent film starts to deteriorate after some measurements. On the other hand, the ProtoBili measurement procedure includes a phantom adapter with a perfectly matched cover, allowing device positioning with higher stability and without any pressure requirement to trigger the measurements.

Table 1 presents Lin’s CCC between the ProtoBili and JM-105 measurements for both translucent films. CCC values close to 1 indicate strong agreement between the two devices, with the 75% translucent film showing better concordance than the 50% one.

In terms of a comparison of results, in the in vivo study performed by [53], the JM-103 (a bilirubinometer version before the JM-105) presented a bias of 3.04 mg/dL, with a variation of ±3.7 mg/dL and considering a confidence interval of 95%. However, another bilirubinometer using a multi-wavelength approach (Bilicheck) exhibited a bias of 1.28 mg/dL, with a variation of ±3.5 mg/dL and a 95% confidence interval. The custom wearable sensor developed by [54], with IoT functionality, reported no bias and a variation of ±4.6 mg/dL, with a 95% confidence interval. In [44], the comparison of the TSB and JM-103 measurements showed a bias of 1.93 mg/dL, with a variation of ±2.04 mg/dL and a 95% confidence interval. In the same study, the suggested prototype using Machine Learning (ML) presented biases of 0.96 mg/dL, 0.82 mg/dL, and 1.39 mg/dL, depending on the algorithm used, and a variation of ±2.12 mg/dL, ±1.87 mg/dL, and ±2.1 mg/dL, respectively. Lastly, the study by Mazrah Mohamed et al. [55] compared TSB measurements with JM-105 measurements, and the mean difference for the forehead was 0.58 mg/dL, with a variation of ±4.7 mg/dL (95% confidence). Similar results were obtained for the sternum.

Table 2 summarizes the bias and precision values obtained in this work for the ProtoBili and the JM-105, along with the results from the aforementioned studies for comparison. Although each study population is diverse, the results from [44] indicate that ML is promising, with the highest precision among the above-mentioned studies. The values obtained by the ProtoBili are clinically relevant, considering that the American Academy of Pediatrics (AAP) considers that a non-invasive bilirubinometer should have an accuracy lower than 3 mg/dL for concentrations below or equal to 15 mg/dL in order to be used for jaundice monitoring of newborns [47].

Considering the above results as one important validation step for the ProtoBili, as well as the capacity of the device to predict its skin coupling status [30], the potential of the ProtoBili for tracking increases in the bilirubin rate in newborns is very promising and will be further investigated for risk assessment and guidance on the treatment initiation in future experiments [15].

## 4. Conclusions

The mini-spectrometer sensor AS7341 for jaundice monitoring in newborns achieved significant results in the in vitro analysis. The ProtoBili and its capability for remote monitoring via BLE can improve the management of jaundice in newborns outside hospitals or reduce clinicians’ workloads within hospitals. Furthermore, the developed prototype can be used in Low- and Middle-Income Countries, where jaundice incidence is higher and access to proper healthcare is limited [24,25].

The development of phantoms was crucial for this work, as they allowed the simulation of different skin tones and a performance evaluation of the jaundice prototype in a controlled environment. More specifically, the use of phantoms with different melanin and bilirubin characteristics was fundamental for calibration and for establishing the posterior calculation process. The in vitro validation of the ProtoBili demonstrated the potential of the proposed approach for bilirubin monitoring in the 0–15 mg/dL range, which is the most relevant range for newborn jaundice management [47].

The next steps of this work are to extend the sensor analysis to in vivo measurements with newborns. The idea is to validate the device-coupling status prediction in newborns, evaluate the device’s wearability and comfort during extended use, and generate a database for future improvements in bilirubin calculation. In addition, the combination of this sensor with other sensing modules, such as temperature and PPG sensors, in the same WBAN network will be investigated to develop a sensor fusion approach for the information supplied to clinicians.

## Figures and Tables

**Table 1:** Concordance correlation coefficient between the JM-105 and the ProtoBili.

Description	CCC
JM-105 (75%) and ProtoBili	0.95
JM-105 (50%) and ProtoBili	0.89

**Table 2:** Performance comparison of bilirubinometers.

Source	Bias (mg/dL)	Precision (mg/dL)
JM-105 (75%)	−2.04	±4.07
JM-105 (50%)	−3.14	±4.91
ProtoBili	−0.94	±2.09
JM-103 [53]	3.04	±3.70
Bilicheck [53]	1.28	±3.50
Custom TcB [54]	0	±4.60
JM-103 [44]	1.93	±2.04
Custom TcB [44]	0.82	±1.87
JM-105 [55]	0.58	±4.70

## Data Availability

The data presented in this study are openly available on GitHub at https://github.com/f-crivellaro/multi-spectral-database (accessed on 27 October 2025).
